# Stakes of Assessments in Residency: Influence on Previous and Current Self-Regulated Learning and Co-Regulated Learning in Early Career Specialists

**DOI:** 10.5334/pme.860

**Published:** 2023-06-16

**Authors:** Indra Ganesan, Breana Cham, Pim W. Teunissen, Jamiu O. Busari

**Affiliations:** 1 Department of Pediatrics, Kandang Kerbau Women’s and Children’s Hospital, Singapore; 2 Department of Genetics, Kandang Kerbau Women’s and Children’s Hospital, Singapore; 3 School of Health Professions Education (SHE), Faculty of Health, Medicine and Life Sciences, Maastricht University and Department of Obstetrics & Gynecology, Maastricht University Medical Center, Maastricht, the Netherlands; 4 Department of Educational Development & Research, Faculty of Health, Medicine & Life Sciences (FHML), Maastricht University, Maastricht, The Netherlands; 5 Department of Pediatrics and HOH Academy, Horacio Oduber Hospital, Dr. Horacio E. Oduber Boulevard #1, Oranjestad, Aruba

## Abstract

**Introduction:**

Assessments drive learning but the influence of the stakes of the assessments on self-regulated (SRL) during and after residency are unknown. As early career specialists (ECS) must continue learning independently, the answer to this is important as it may inform future assessments with the potential to promote life-long learning after graduation.

**Methods:**

We utilized constructivist grounded theory to explore the perspectives of eighteen ECS on the influence of stakes of assessments within residency on their SRL during training and in current practice. We conducted semi-structured interviews.

**Results:**

We initially set out to examine the influence of the stakes of assessments on SRL during residency and after graduation. However, it was apparent that learners increasingly engaged with others in co-regulated learning (CRL) as the perceived stakes of the assessments increased. The individual learner’s SRL was embedded in CRL in preparation for the various assessments in residency. For low-stakes assessments, the learner engaged in less CRL, taking less cues from others. As stakes increased, the learner engaged in more CRL with peers with similar intellectual level and supervisors to prepare for these assessments. SRL and CRL influenced by assessments in residency had a knock-on effect in clinical practice as ECS in: 1) developing clinical reasoning, 2) improving doctor-patient communication and negotiation skills, and 3) self-reflections and seeking feedback to deal with expectations of self or others.

**Discussion:**

Our study supported that the stakes of assessments within residency reinforced SRL and CRL during residency with a continued effect on learning as ECS.

## Introduction

Self-regulated learning (SRL) is an essential skill that residents need to develop as it contributes to academic success during residency [1] and professional success after [2,3]. However, it remains to be understood how graduating residents will adapt their SRL after transitioning into independent practice [4]. SRL empowers learners to take ownership of their learning and represents a fundamental prerequisite to life-long learning. SRL involves a cyclical process in which individuals use metacognitive processes to plan, execute and evaluate strategies to achieve their learning goals [5,6]. After graduation, residents should be equipped with skills to continuously engage in SRL to keep up with new clinical knowledge and maintain mastery of their knowledge and skills as practicing physicians [7]. During residency, known triggers of learning in a clinical workplace include patient-related activities, supervisors or peers asking questions that expose knowledge gaps, and the learning objectives of the training programme and related assessments [8,9,10,11]. Although it is known that assessments significantly impact what learners focus on [12,13], the regulation of learning as determined by the stakes of assessments during residency and how this influences the continuous adaption of their SRL after transition into clinical practice is unestablished.

Self-regulated learning theory offers an understanding of how a person becomes a lifelong learner, as the prevailing skills required for SRL are also crucial for effective lifelong learning [10,14]. Zimmerman defines SRL as three cyclical phases of forethought, performance, and self-reflection [15]. In the forethought phase, learners analyse the task, set goals, and think about strategies to reach their goals. In the performance phase, they utilize various task strategies such as help-seeking and environment structuring to execute their learning tasks and seek the motivation to complete them on time. Self-reflection is the final phase and constitutes self-evaluation and self-judgement of the solutions applied towards the final goals. For future tasks, learners may choose to use the same successful strategies or employ new strategies to obtain better results. Self-reflection, in turn, influences forethought in subsequent tasks, which contributes to the model’s cyclical nature [15]. A recent study highlighted that SRL is context-dependent in a clinical workplace by showing that novice medical students’ SRL depends heavily on peers and residents to clarify their roles and set external learning goals [16]. However, SRL in experienced medical students had less peer influence as they had intrinsically set their own goals but sought necessary feedback from residents and consultants to become the type of doctor they wanted to become [16].

Assessments are designed to support and evaluate learning. Formative assessments are lauded as an ‘assessment for learning’ and for their role in developing SRL. The feedback provided to learners enables them to adjust their learning to achieve current and future intended learning outcomes [17]. For formative feedback to enable development of SRL, it must have clear qualitative information on current performance, well-articulated goals on desired learning outcomes, and suggestions on how to close the gap to improve subsequent performance [18]. In contrast, summative assessments are characterized as an ‘assessment of learning’ that evaluates knowledge and proficiency and are often associated with a high-stakes consequence such as progression to the next level of training or final certification [19,20]. The design of the summative assessment heavily influences the learner’s learning behaviors. Summative assessment questions that require learners to apply knowledge and critical thinking to construct their answers invoke a deeper learning approach, whereas questions assessing facts and lists promote memorization [19]. Programmatic assessments were designed to maximise learning opportunities that arise from feedback from multiple formative assessments [21,22].

Schut et. al. highlighted the need to consider how learners perceive the stakes of an assessment instead of focusing on a formative versus summative dichotomy. Even in the formative milieu of programmatic assessments, learners perceived low-stakes assessments as high-stakes if it impeded progression. The perceived stakes of the assessments were lowered if they were given opportunities to exercise control and influence the outcome of the assessments [13]. As “the stakes are in the eyes of the beholder”, the influence of the stakes of assessments on the development of SRL during residency and into practice is unknown [13]. This information may inform the design of future assessments while accounting for the perceived stakes of the assessments to the learners to promote effective SRL during residency but may also promote life-long learning after graduation. This study aimed to explore the perspectives of early career specialists who have already transitioned into clinical practice to answer the research question: How did the stakes of assessments within a residency program influence early career specialist’ SRL during their residency and in current practice?

## Method

We used constructivist grounded theory (CGT), a qualitative methodology suited to explore the social processes of SRL of early-career specialists (ECS) in current clinical practice [23]. We chose CGT as we hoped to add a theoretical understanding to the existing SRL theory on the aspects of how the stakes of the assessments influence a learner’s SRL. This study was approved by the Singapore Health Services (SingHealth) ethics review board (CIRB Ref 2020/2736).

### Postgraduate medical education assessment context

This study was conducted with early career pediatricians (ECPs) at Kandang Kerbau Women’s and Children’s Hospital in Singapore. There were two summative assessments in the 6-year pediatric residency programme. A midpoint assessment, the Membership of the Royal College of Physicians and Child Health (MRCPCH) examinations which tested proficiency in obtaining clinical history, performing physical examination, management planning and communication skills. The final exit assessment was a structured oral viva-voce examination which evaluated maturity in clinical reasoning, diagnosis, and management. Formative assessments were embedded throughout residency. In mini-clinical examination (mini-CEX) assessments, residents were given immediate feedback on observed clinical encounters. Case-based discussions (CBDs) involved a comprehensive written exploration of an approach to a clinical problem with literature review of best practices. The exit assessment and MRCPCH examination were high-stakes by design, and mini-CEX and CBD were low-stakes. The reflective write-up on their self-reflections of a clinical encounter were mandatory low-stakes assessments that were submitted annually.

### Sample

We recruited ECPs as they experienced SRL in a new role similar to other ECS in their transition into independent practice. These ECPs graduated from a pediatric residency program with both high-stakes and low-stakes assessments, which was ideal for answering our research question. In Singapore, high-stakes assessments are a prominent feature of the public education system and university programmes and entry into medical residency programs are extremely competitive. We interviewed 18 participants whom we defined as ECPs: practicing pediatricians who graduated from residency within the past five years. They were in a position to inform us on their current learning strategies, but remained close enough to their residency training, thus retaining the ability to make parallel comparisons between their current and previous learning strategies whilst in residency training and provide insights on how residency program assessments may have influenced current SRL. As we were interested in the effects of stakes of assessments in residency training on SRL and its application during clinical practice when participants settled in their new role as ECPs, we deliberately chose to sample up until five years after post-residency training, similar to sampling methods in other studies on ECS [2,24,25,26]. Transition literature has indicated the immediate post-transition period to be an emotionally unnerving experience due to the plethora of new non-clinical skills that these ECS were expected to perform [27,28]. The ECPs were purposively sampled to ensure variety in gender, subspeciality training and years of training post-graduation. Of the 18 participants, 11 were female and 13 were in subspeciality training and rest were in general paediatrics. The mean post-graduation period was 1.91 ± 1.27 years, and their mean age was 34.4 ± 2.0 years. These ECPs continued to practice in the same hospital as their residency program.

### Interview approach

During interviews, we asked the ECPs to explore their current SRL. Subsequently, the ECPs were asked to reflect on their residency training years using “how”, “what” and “why” questions to explore their previous SRL. The initial interview guide was broad, exploring their SRL as an ECP and their SRL during residency. When a topic was raised during the interview, it was explored further. For example, when a participant discussed learning new skills as an ECP such as managing a clinical team, learning to prioritize to function efficiently, or assessments during residency, this was subsequently explored using SRL phases. We asked them to describe the forethoughts that they considered in formulating and addressing their learning goals, then what were the processes that they had done to achieve the learning task to achieve their intended goal. Participants were also asked to describe their reflections, on how the process had gone so far, how they monitor their progress, and what their considerations were for future learning tasks. Following the eighth interview, the interview guide became more focused on (i) preparation for assessments (focusing on the stakes of the assessment for the learner), (ii) SRL strategies used to prepare for assessments, and (iii) SRL strategies as they continue to learn as an ECP. Even though we were particularly interested in the influence of stakes of the assessments on their SRL during residency and if or how this may have shaped their current SRL practices, we took great care not to lead the participants into seeing links between assessment in residency and current learning by not asking leading questions. Semi-structured interviews lasting 45–70 minutes were conducted by IG, and transcribed verbatim with identifying data removed.

### Data analysis

Data collection and analysis followed an iterative process consistent with CGT methodology [23] to inform new data collection through exploration of generated themes with new interview probes. IG and BC independently coded transcripts 1–3 using line-by-line coding to identify SRL concepts in linking the learning experience during residency. Subsequently, we analysed the transference of SRL into clinical practice as an ECP. We used ATLAS.ti, a qualitative research software, to code the data and develop a code book after the third transcript. All team members attended multiple meetings to discuss the findings. At team meetings, we defined the themes of interest that were generated by reiterative engagement derived from the codes and coding process in the data set. After analysis of six transcripts, the participants described their SRL strategies when preparing high-stakes assessments with the pronoun “we” utilizing sentences like we would try out some questions, we would practice with each other, we would judge our performance, we would then try to reach the benchmark by reading around the topic and seeking help from supervisors. In line with CGT reiterative analysis of the data, we unpacked the term “we” to realize that these participants were describing their co-regulated learning (CRL) in which they were self-organizing themselves into small study groups in preparation for high-stakes assessments. This also involved taking cues from peers and supervisors to regulate their own learning (CRL). We probed further on the concepts of co-regulated learning (CRL) in our participants utilizing the definition of CRL that describes how the learners’ social interactions with others in the environment influence the learners’ cognitions, emotions, and motivation for learning [29]. We sought understanding on how the stakes of previous assessments influenced participants’ SRL and CRL during residency. Subsequently, we examined whether links could be drawn from their past insights to their current SRL and CRL in clinical practice. Aligned with CGT methodology, we co-constructed our knowledge through our interactions as researchers with the ECPs as participants.

### Reflexivity

IG is a medical educator, paediatrician and a PhD student in medical education who had observed the residency journeys of these ECPs. IG underwent training in a residency program with both high and low stakes assessments and was aware of how this experience modulated her learning strategies in response to the stakes of assessments, and would have potentially influenced her interpretation of the study data. Therefore, IG used memos and field notes to capture her views, and was conscientiously checking to ensure that the initial coding and subsequently analysis remained close to the participants’ views. This was also reviewed by BC, a researcher and a clinical genetic counsellor, who was not directly involved in the participants’ training. PWT is medical educator (PhD) and a practicing obstetrics and gynecology physician, and JB is a medical educator (PhD) and a pediatrician. PWT and JB brought their perspectives from their specialities and active involvement in residency training programs that primarily employ formative assessments and a few high-stakes assessments during residency. They were initially critical of the potential effects of learning in very high-stakes assessments and we reflected on this aspect as a team during this project.

## Results

The participants reported that the degree to which they engaged in behaviors to improve their prioritization and efficiency of learning increased as they perceived assessment stakes to be high. The lower stakes assessments mainly allowed them opportunities to consolidate their learning experiences by repeated practice with authentic clinical encounters. We present our findings in two interrelated aspects. First, how the stakes of the assessments (high-stakes and low-stakes) influenced learning in residency and second, how their studying and learning behaviors during residency morphed into different ways of learning as an ECP.

### Assessment Stakes Influenced Learning in Residency

#### Hig-Hstakes Assessments

Participants perceived the stakes of the midpoint and exit examinations to be high as was intended by their program. Across the data, participants spoke of the following underlying goal of their SRL strategies: to acquire and organize the most relevant, practical and applicable knowledge most efficiently in the least amount of time. As these assessments affected their career progression, they reported a significantly increased intensity and time allocated in preparation for these assessments. They sought numerous ad-hoc tutorials with faculty supervisors and clarified any misconceptions or conceptual doubts. They also reported a high state of anxiety about the outcome of the high-stakes assessments as there was a significant fear of failure due to the subsequent consequences to their careers and life: “I put six months of my life on hold in order to prepare” (P5). Hence, they sought ways to improve their efficiency in their learning process by studying in self-initiated peer groups, actively seeking high-quality feedback to monitor and improve their performance, and seeking opportunities to practice clinical reasoning. These study groups of three to five members were formed by participants choosing peers with similar learning approaches and academic calibre: “working towards the same goal, the high-stakes exam.”(P9). The co-regulated learning that occurred in these peer study groups ensured that residents remained focused in their preparation of the high-stakes assessments: “you don’t want to be the one who is just a freeloader and not contribute. So I think that the main thing about it is peer pressure.”(P9). These peer study groups offered multiple other benefits: “Studying in a group allows me to practice verbalizing my thoughts. Your peers can observe your insufficiency and give you feedback immediately on how to phrase your answers better. It is having someone to always countercheck your answers.” (P7).

#### Low-stakes Assessments

Participants perceived low-stakes assessments, such as mini-CEX and CBDs, as an on-going formative discussion between themselves and their assessors. These served to identify gaps, increase comprehension, and prompt reflection on what they learnt longitudinally throughout the residency: “I treat it [mini-CEX/CBD] more like a dialogue session” (P9). As mini-CEXs were perceived as ad-hoc low-stakes assessments, they did not invest much time in preparation: “don’t want to do poorly… [but] I will not go out of my way to prepare for a mini-CEX” (P16). Contrary to the preparation for high-stakes assessments, participants felt that it was inefficient and too time consuming to organize peer study groups to prepare for these low-stakes assessments: “for the low stakes, it would not involve too much preparation beforehand… [not] very useful to study in a group, because everybody is in different stages of their formative assessment.” (P9). However, one participant felt that the stakes in mini-CEX would be elevated with a stern assessor, so there was a deliberate selection of assessors based on their perceived approachability in order not to: “get bashed with your mini-CEX examination” (P7).

### Knock-on Effect of the Assessment Program on Learning as ECPs

Participants experienced that stakes of the assessments drove their SRL and contributed to their performance as ECPs. Building on participants’ descriptions of their self-regulated and co-regulated learning during residency, we identified three areas of connection to their learning as ECPs: 1) Developing clinical reasoning, 2) Improving doctor-patient communication and negotiation skills, and 3) Self-reflections and seeking feedback to deal with expectations of self or others. The links between the influence of stakes of assessments on learning during residency and how this has subsequently influenced their learning as ECPs are described below.

#### Developing clinical reasoning

Given the broad syllabus for the high-stakes exit assessment, participants had to prioritize their learning of: “clinical problems that were commonly encountered but also rare problems that were life-threatening which were important to recognize” (P17). They described the need to refine the mental representation of clinical problems, being cognizant of conflicting evidence and considering alternative diagnosis in their clinical reasoning. Now as ECPs, this skill had helped to reduce availability bias by identifying salient red flags in clinical information that may indicate a less common underlying diagnosis for a common presenting complaint: “think about all the red flags and think about all the pitfalls that could happen. As a practicing pediatrician, you’re not going to assume that every other kid with fever is just having influenza, you need to be able to find out who is a septic child.” (P2)

The participants reported that the design of the high-stakes exit examinations in which they were expected to problem solve in a short time period promoted efficacy in the synthesis and analysis of clinical information under time pressure. This skill was applicable to them in their current practice as ECPs in clinical reasoning and decision making during busy ward rounds.

“In an exam situation, you are forced to think fast, analyze all these problems very quickly. As a consultant, you have a lot of patients in the ward, how to pick up the important bits to focus on. So, I can function in faster, a more comprehensive, logical manner so I can deliver more holistic care to the patients.” (P4)

#### Improving doctor-patient communication and negotiation skills

i) Doctor-patient communication. In residency, participants were taught a communication framework when engaging patients and family. In co-regulated learning study groups in preparation for high-stakes assessments, participants practiced difficult communications about dealing with an angry parent, giving bad news or helping parents to deal with a life-limiting condition.

As an ECP, they gradually realised that acquiring the soft skills of communications was far more complicated and nuanced, often going beyond the previously learnt communication framework in preparation for their assessments. The metalevel of communication that they learnt was the need to consider the perspective of patients and other stakeholders, to signpost the discussion so that the other person was prepared for the next stage of the conversation, and deliver the information in smaller comprehensible chunks using audio-visual processing aides.

ii) Negotiation skills. Although participants reported many benefits of group study, a downside was difficult negotiations with peers on differences in opinions which were more challenging in a high anxiety state due to the upcoming high-stakes assessments: “in a stressful situation, people tend to be a little bit more highly strung” (P9). In attempting to resolve these differences in opinions, they had resorted to use important communication skills of perspective taking, finding solutions, compromising, and finally, if all fails then to respectfully “agreeing to disagree” (P10). One participant felt that communicating difference in opinions with senior colleagues was a daunting process as an ECP, and hence was grateful for the opportunity to practice voicing differences in opinions in their study group in residency:

“When we study in a group, we try to manage interpersonal relationships. By doing this communication of differences in opinions in the study group may be easier when starting off with peers because you don’t feel threatened [as] much. Then, you could learn these skills and then apply it into your daily interaction and communication with seniors.” (P7)

As ECPs, the participants found that they needed to negotiate and work together for a patient even when there were differences in opinions amongst clinical teams. This art of negotiation required metacognition of recognizing perspectives of the other parties in relation to their own and compromising when necessary:

“First, I will try to understand what is the point of view behind what the other person is proposing. I would often end up agreeing if it is reasonable. But if it is something that I strongly feel against, then I would want to respectfully communicate my concerns directly to the person. Then we will continue to discuss and try to come to a mutual agreement.” (P12)

#### Self-reflections and seeking feedback to deal with expectations of self or others

Participants acknowledged that their self-reflection in reflective write-ups were superficial as these exercises were deemed to be low-stakes. In comparison, participants engaged in deeper self-reflection to improve their performance in the midpoint and final high-stakes assessments. One such method was seeking high quality feedback from supervisors and peer residents to detect diagnostic or performance errors. This mark-up in self-reflection was attributable to the participants fear of failure or previous experience failing a high stakes examination.

“the preparation [for the exit examination] was very intense as the outcome of the exam may not be what you expect it to be. So, then it really involves a lot of reflection, sometimes you get very general feedback, I don’t think that’s helpful, because someone needs to take you through the process or help you to reflect on your errors because you don’t see it [your deficiency]. If you don’t have insight, you can’t move forward.” (P2)

Similarly, ECPs reported not engaging in deep self-reflection on a daily basis: “on a regular day, I don’t really just reflect like, you know, today what did I learn, things like that.” (P1). In contrast, after a negative experience, they reported going through a deliberate process of self-reflection where they analyzed the circumstances leading up to the encounter and reflected by themselves, with peer ECPs, or seniors on whether any actions or behaviours that could have modified or improved the outcome:

“the reflection comes when something bad happens, like a death or negative feedback from a peer or senior or a complaint about me. So, in these moments, I will really reflect for some time on the circumstances around it, there’s always something that you can get out of it and think how I can better do certain things.” (P1)

## Discussion

This study explored how ECPs perceived the stakes of assessments in residency and their potential influence on current SRL behaviors. Our findings show that the stakes of the assessments play an essential role in the regulation of learning (both SRL and CRL) during the residency, which has a continued longitudinal effect on learning after graduation from residency.

We initially set out to examine the influence of the stakes of assessments on learners’ SRL. However, it soon became apparent that individual learners increasingly engaged with others in a CRL process in tandem with the perceived increase of stakes in the assessments. Our findings corroborate the idea of SRL being embedded in co-regulated learning [16,30]. Moreover, our results help to expand the current concepts of SRL and CRL in medical education. Historically, research in this domain has focused on the perspective of the individual regulating his or her own learning (SRL) [5,31]. In SRL, the individual sets the goals, engages in processes to execute learning and then monitors one’s progress in a cyclical manner onto the next learning task [31]. A recent study by Bransen et. al. highlighted that learning goes beyond the self to others as a shared regulation of learning (CRL) in their learning environment [16]. The learners were described to be taking cues from others to optimize their learning in clinical clerkship [16,32,33].

**Figure 1:**
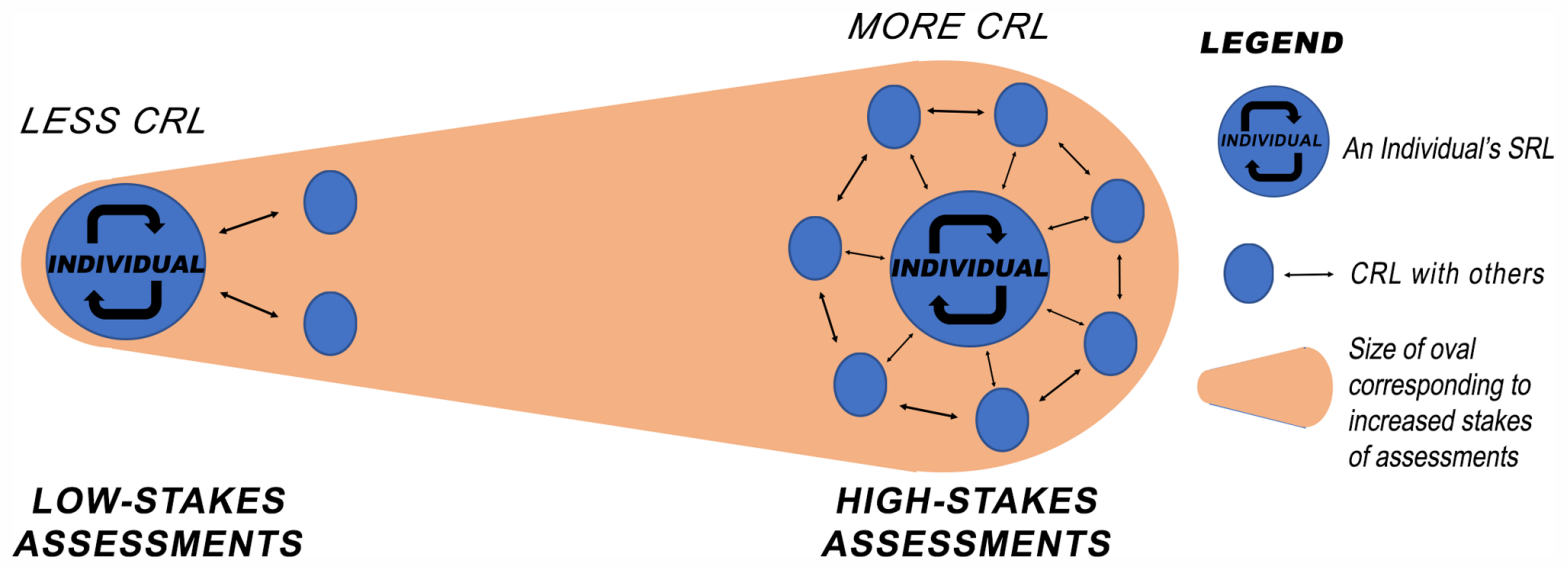
Conceptual model of the SRL – CRL in relation to stakes of the assessments.

Our study adds that co-regulated learning is constantly occurring. Learners take varying amounts of cues from others to regulate their learning [32,34,35]. By studying how the stakes of the assessments influences the regulation of learning, we found the amount of CRL activities differ in preparation of low-stakes in comparison to high-stakes assessments. From the perspective of an individual learner, as the stakes of the assessment increase, the amount of CRL activities correspondingly increase. In preparation for low-stakes assessments, there is less CRL. In Figure 1, there is a low amount of CRL between an individual (large blue dot) and others (smaller blue dots) when the stakes are low. As the stakes increase (oval wedge background), the extent of CRL correspondingly increases between the individual and others). In both situations, the learner engages in SRL (circular arrows in blue dot of the Individual). Even though the individual learner may study in a group or engage with supervisors in preparation for high-stakes assessments, it is the onus of the individual learner to pass the assessment by being well-versed with the relevant assessment syllabus. So, the high-stakes assessments drive SRL in an individual learner. In response to low-stakes assessments, learners go through all the phases of SRL with a few others regulating their learning. This is driven by learning needs to fulfil knowledge gaps and occurs longitudinally throughout residency. The residents define their knowledge gap in the forethought phase and then choose the appropriate case to write-up or to perform a physical examination on. Subsequently, they seek credible feedback from supervisors after case-based discussions or the mini-CEX assessments, and then judge if the knowledge gap is sufficiently closed. Our study therefore corroborates that SRL in residents are externally driven by an upcoming assessment [19,36,37] and adds this happens regardless of the stakes of the assessments.

Participants felt that engaging with peers with similar intellectual calibre was an important pre-condition for engaging in CRL in improving their performance in the final high-stakes assessments. However, the students in an introductory course in computer programming showed slightly lower performance in the final assessment when they engaged in higher co-regulation [38]. The authors themselves were surprised by these findings as the majority of these students had report positive beneficial effects of studying in a group, similar to our findings [38]. We postulated that the perceived beneficial effect of increased co-regulation in our participants was due to the design of the final exit assessment (oral viva-voce) which rewarded repeated practice opportunities to articulate thought processes [19].

Aligned with Bransen et. al’s findings, medical students over time increasingly take control and engage with others in co-regulated activities to support their learning [16]. Our study indicates that a longitudinal development of the regulation of learning, in preparation for assessments, extends beyond residency into clinical practice as early career specialists. The learning in preparation for assessments in the domains of communication and clinical reasoning, and how they continue to learn as early career specialists, are explicit. In residency, residents are taught clinical reasoning and communication methods in which they continue to develop and refine as they transition into practice [39]. Our study adds that there are valuable indirect links in the art of negotiation when addressing differences in opinions amongst peers in a study group that is applicable to their current practice when co-managing patients with different specialities as early career specialists. Engaging in deep self-reflection to identify diagnostic or performance errors using high-quality feedback to enhance performance in the high-stakes assessment is another example of an implicit link to self-reflection when dealing with a an unexpected and negative outcome in their current practice as early career specialists.

### Limitations and implications for future research

First, due to the study design, it was inevitable that both the topics of assessments in residency and their current SRL were discussed. However, we took great care not to suggest to participants the possible links between assessments in residency and their current SRL. We allowed participants to speak freely about their experiences on SRL during residency, SRL in preparation for assessments, and current SRL in clinical practice. We avoided directed questions like “Did the stakes of assessments in residency influence your current SRL now?” as these types of close-ended questions tend to invoke a yes or no response thereby limiting the opportunity to explore the breadth of the discussion. Second, this study was deliberately conducted in a context with both high-stakes and low-stakes assessments. Hence, our findings of the effects of assessment stakes on learning later in the physician’s careers could be different to other assessment programs. Third, other processes independent of assessments, such as self-reflective practice when faced with challenging clinical situations or personal curiosity, may have contributed to the development of SRL in ECPs.

Theoretically, SRL could be an entirely self-initiated and intrinsically motivated process [5]. However, because assessment of competency occurs at all levels of medical training, SRL and CRL are also subject to extrinsic sources of motivation such as assessments. Furthermore, our findings demonstrated that assessments can influence the SRL and CRL of trainees, hence it is important that educators explicitly address this factor in their assessment design. Sandars, and colleagues have recently similarly suggested that we need to provide learners coaching and support to develop co-regulated learning behaviors, building upon the effects of the assessment programs [40]. Our findings indicate the learners choose to engage in CRL with supervisors to clarify conceptual understanding and to seek feedback on ways to improve their performance. So, supervisors should be aware of their important role in supporting this learner initiated SRL and CRL. One area of future research is to study the complex dynamics in which co-regulated learning is formulated amongst learners. In programmatic assessments, formative assessments that focus on articulating thought processes and reasoning strategies may promote CRL among the amongst learners and their supervisors. However, this will need further investigation.

## Conclusion

Our study supports the idea that the stakes of assessments within residency program reinforces SRL and CRL behaviors with a continued longitudinal effect on learning after graduation from residency. Additionally, as the stakes of the assessments increase, the amount of CRL between learners of similar intellectual levels and with their supervisors also increases.

## Additional File

The additional file for this article can be found as follows:

10.5334/pme.860.s1 Appendix. Interview Guide.
