# Supplementary material for: Stakes of Assessments in Residency: Influence on Previous and Current Self-Regulated Learning and Co-Regulated Learning in Early Career Specialists
Source: Perspect Med Educ. 2023 Jun 16;12(1):237–46. doi: 10.5334/pme.860 (PMC10275342; doi:10.5334/pme.860)
Supplement: Appendix. — Interview Guide. [file pme-12-1-860-s1.pdf]

Appendix: Interview Guide

Introduction

Hi, good morning/evening. The reason why you are invited for this study is because you are an early career paediatrician who has recently graduated from the residency program. I would like to explore about your learning during the Residency program and how that has influenced you now as a practicing paediatrician on how you continue to learn and develop yourself. At any point, if you're uncomfortable with the interview process, please let me know. This interview will be audio recorded so that it can be transcribed. Once the verbatim is transcribed, any identifying information will be removed. Do you have any questions? (pause) If not, I will just continue with the interview. Is that okay with you?

Date:

When did you graduate from the pediatric residency program? How many years ago was that?

Age: years

Gender:

Subspeciality: yes/no

Questions

1. During the residency program, you learn your profession, you develop into the pediatrician that you are now. Now, functioning as an early career pediatrician, what are the skill sets that you are learning to function to do a day-to-day work?
   Further probes for skills learnt:
   - What triggers your current learning goals?
   - How did you go about achieve these learning goals? What are your learning processes?
   - How did you monitor these learning process?
   - What were your self-reflection on these learning moments?
   - Did you carry forward any of these to the new learning tasks?

2. Okay, so, can you tell and tell me about an interesting case recently that you were involved in that describes all these learning processes as an early career pediatrician.

3. During your residency, you learned your profession, and have become the pediatrician that you are now, can you please describe the learning processes during your residency?
   - What triggered your learning goals?
   - How did you go about achieve these learning goals? What were your learning processes?
   - How did you monitor these learning process?
   - What were your self-reflection on these learning moments?
   - Did you carry forward any of these to the new learning tasks?

4. Further probes when a participant discussed assessments as trigger for learning.
5. In the later interviews: If participant does not mention about assessment being a trigger for learning, then ask about the role of assessments.
   Initially, can please describe all the assessments that you needed to do in your residency training?
   How will you describe the stakes of each of these assessments, on what it means to you?
   Please describe were your consideration in deciding your learning goal. What were the first step and subsequent steps that you undertook to plan and prepare for these assessments? What were the processes undertaken to achieve your intended goal? How did you monitor your progress? What were your reflections on how the processes had gone so far? What were your considerations for future learning task?

Subsequent interview questions

Co-regulated learning

1. Who is the “we” that you mentioned in preparation for the Exit exams?
2. As you were saying, that there's a group of you studying together. Can you describe more about the group dynamics?
3. When is the group formed?
4. How does choosing the group members come about?
5. How do you decide on the number of study group members?
6. How do you all learn from each other during this group learning?
7. How do you decide what you are going to study in the group?
8. How do you learn when studying in a group? How is this different from studying individually?
9. What are the advantages of studying in a group? What are the barriers that you faced studying in a group?
10. So, you said that by study in group that you learnt how to …… Probe further on what the participant mentioned (e.g. develop interpersonal skills/ engage in teamwork, so what you mean by develop interpersonal skills/ engage in teamwork?)
